# Case Report: A case of ovarian carcinoma manifesting with chest tightness as the initial clinical presentation accompanied by marked elevation of pleural effusion amylase levels

**DOI:** 10.3389/fonc.2026.1616309

**Published:** 2026-01-20

**Authors:** Guo-Yu Ou, Yin-Chuan Zhu, Kun Li, Cheng-Jie Li, Xin Yi, Qiu-Bin Wan, Hai-Lan Shen

**Affiliations:** 1 Department of Laboratory Medicine, Fengdu General Hospital, Chongqing, Fengdu, China; 2 Department of Laboratory Medicine, The First Affiliated Hospital of Chongqing Medical University, Chongqing, China

**Keywords:** amylase, chemotherapy, diagnosis, ovarian cancer, pleural effusion

## Abstract

This article reports a case of ovarian cancer presenting with chest tightness as the initial symptom, accompanied by markedly elevated pleural fluid amylase. A middle-aged female patient sought medical attention for “chest tightness and wheezing” as her primary complaints. Imaging revealed a massive pleural effusion with significantly elevated serum and pleural fluid amylase levels. Pleural fluid cytology staining and ovarian tissue pathology confirmed the diagnosis of high-grade serous carcinoma of the ovary. Subsequently, she underwent open abdominal cytoreductive surgery for ovarian cancer under general anesthesia, which included total hysterectomy, bilateral salpingo-oophorectomy, and omentectomy. Adjuvant chemotherapy with paclitaxel and carboplatin was administered two weeks after surgery. Amylase levels returned to normal after four weeks. This case suggests that in female patients with unexplained abnormally elevated serum and body fluid amylase levels, clinicians should remain vigilant for the possibility of ovarian cancer. Amylase may serve as an auxiliary diagnostic clue, and its combination with pathological and imaging examinations can facilitate early differentiation. Clinical practice should emphasize the diagnostic value of atypical symptoms and laboratory indicators.

## 1 Introduction

It is rare for amylase—a biomarker of pancreatic injury—to be significantly elevated in ovarian cancer patients. Recently, its potential value in the diagnosis and prognosis of these patients has attracted growing attention (1). In this case, the ovarian cancer patient initially presented with atypical chest tightness. Serum and pleural fluid amylase levels were markedly elevated, and the diagnosis was eventually confirmed by pathology as ovarian cancer. Elevated amylase levels in ovarian cancer have seldom been reported in the literature, which may lead to missed or misdiagnosed cases. This phenomenon therefore warrants clinical attention.

## 2 Case report

A 55-year-old female patient presented to the Department of Cardiology on February 25, 2023, with recurrent chest tightness and exertional dyspnea for half a month, which had worsened over the past week. Physical examination revealed reduced mobility of the left thoracic cavity. The electrocardiogram showed a sinus rhythm. Laboratory tests showed no significant abnormalities, including coagulation function, B-type natriuretic peptide (BNP), and troponin. Ultrasound indicated a large left pleural effusion with left lung atelectasis and a small right pleural effusion. To determine the specific condition and underlying cause of the pleural effusion, we performed an ultrasound-guided left thoracentesis and submitted the obtained pleural fluid specimen for routine laboratory analysis.

The pleural fluid amylase level is markedly elevated at 4995 u/L, with levels significantly higher than those in serum (343 u/L; reference range: 35–135 u/L) and urine (1513 u/L; reference range: 0–1200 u/L). Pleural fluid analysis demonstrated a bloody, turbid appearance with a positive Rivalta test. Mononuclear cells accounted for 89%, polymorphonuclear cells for 5%, and unclassified cells (6%) with enlarged cell bodies, larger nuclei, and deeply stained cytoplasm. Further pathological examination was recommended. Serum carbohydrate antigen 125 (CA125) 120u/mL (reference range: <35u/mL) and human epididymis protein 4 (HE4) 175pmol/L (reference range: premenopausal <76pmol/L, postmenopausal <105pmol/L) are both elevated.

**Figure 1:**
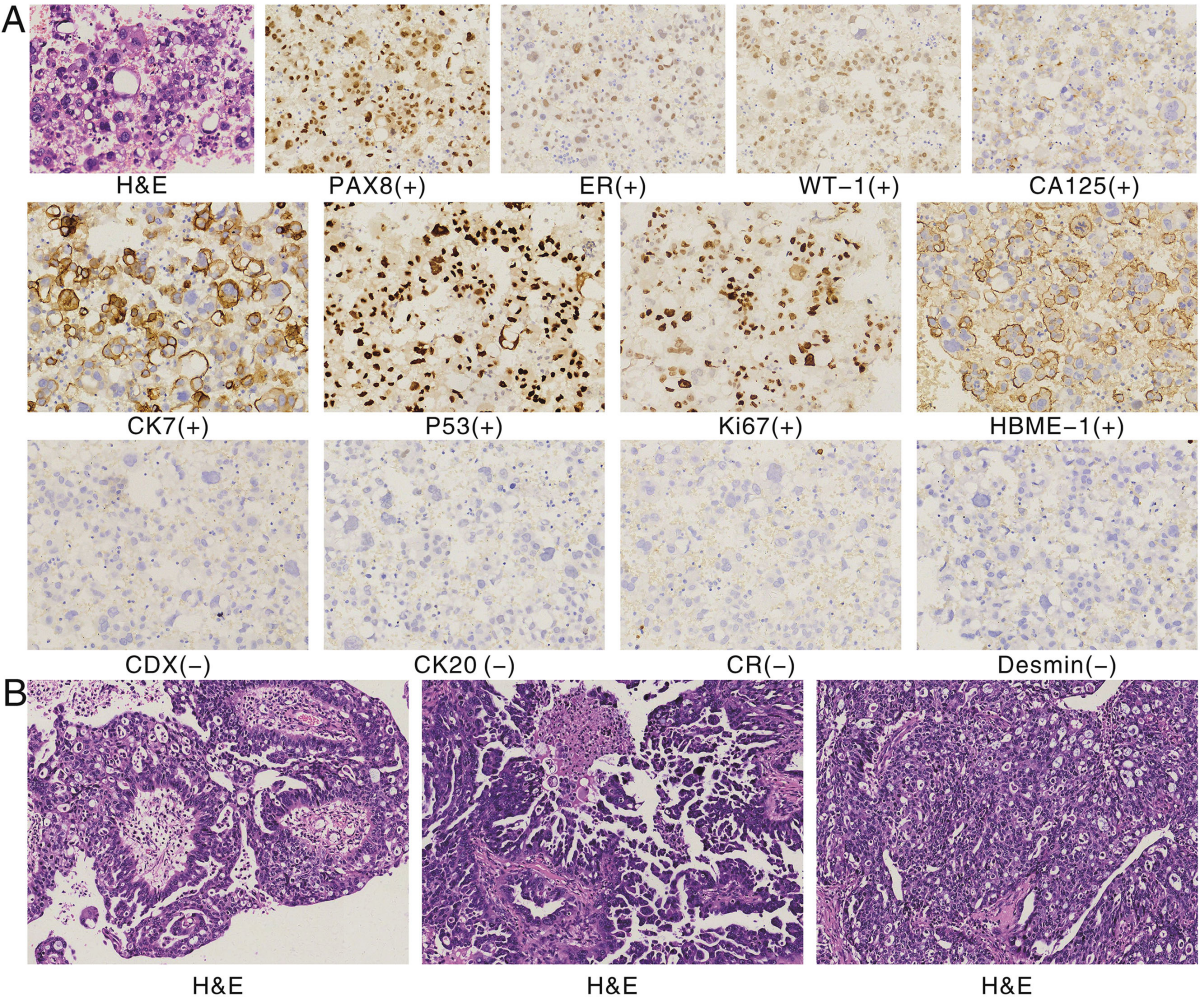
Pathological diagnosis. **(A)** Hematoxylin and eosin (H&E) staining and immunohistochemical staining of pleural effusion cells (×400). The findings are consistent with high-grade serous ovarian carcinoma (HGSOC) per WHO classification. Immunohistochemistry shows positivity for paired box gene 8 (PAX-8), estrogen receptor (ER), and Wilms tumor protein 1 (WT-1); a CK7+/CK20- profile; strong p53 expression; and a high Ki-67 proliferation index. Stains for caudal-type homeobox transcription factor 2 (CDX-2) and desmin are negative, ruling out common colorectal carcinoma and mesenchymal tumors. **(B)** H&E staining of ovarian tissue (×200), showing from left to right: papillary structures, micropapillary structures, and cribriform patterns. Tumor cells display oval morphology, nuclear pleomorphism, hyperchromasia, marked cytologic atypia, and frequent mitotic figures.

**Figure 2:**
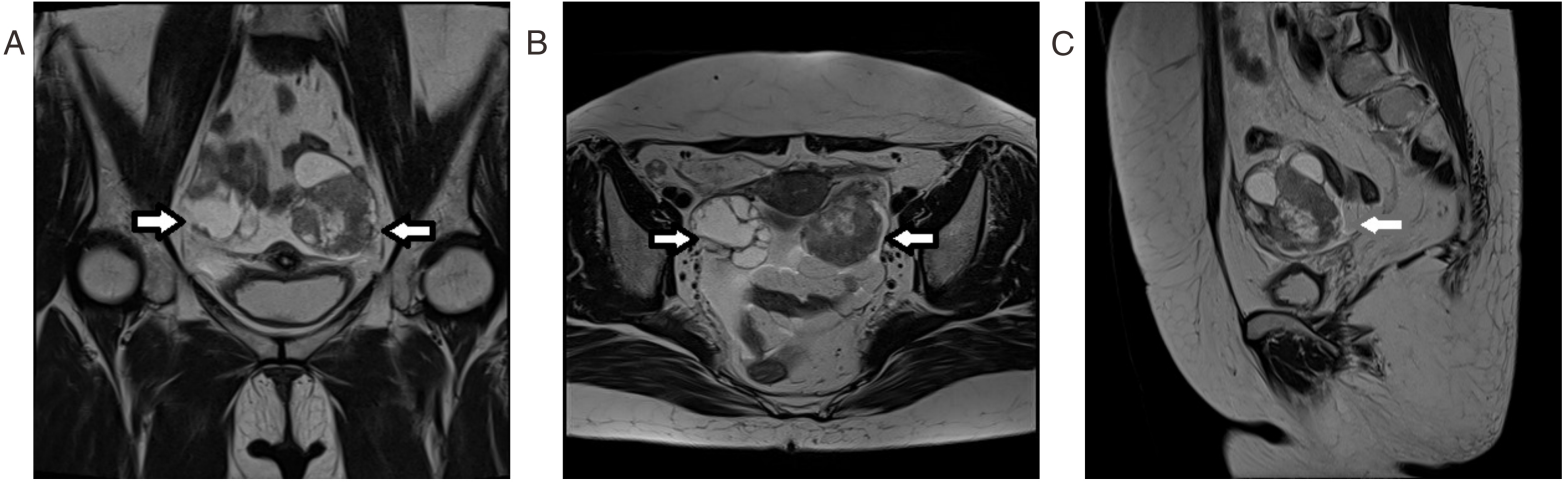
MRI findings of the pelvis:**(A)** Coronal plane, **(B)** Axial plane **(C)** Sagittal plane. Bilateral adnexal regions demonstrate cystic-solid lesions.

Abdominal computed tomography (CT) revealed multiple small retroperitoneal lymph nodes and a large left-sided pleural effusion. Pathological examination of the pleural fluid showed numerous atypical cells. Combined with immunohistochemical staining results consistent with adenocarcinoma, these findings indicated a high probability of ovarian origin. The immunohistochemical profile of the pleural effusion cells was as follows: Positive for paired box gene 8 (PAX-8), estrogen receptor (ER), and Wilms tumor protein 1 (WT-1); cytokeratin 7+/cytokeratin 20- (CK7+/CK20-); strongly positive for tumor protein p53 (p53); weakly positive for carbohydrate antigen 125 (CA125) with a high Ki-67 proliferation index. Negative for caudal-type homeobox transcription factor 2 (CDX-2), desmin, and calretinin (**Figure 1**). Pelvic magnetic resonance imaging (MRI) further confirmed neoplastic lesions in both ovaries (**Figure 2**). The patient was subsequently transferred to the gynecology department. On March 31, 2023, she underwent open abdominal cytoreductive surgery under general anesthesia, which included total hysterectomy, bilateral salpingo-oophorectomy, and omentectomy. The procedure was completed successfully.

Postoperative pathological examination confirmed a malignant tumor, specifically high-grade serous carcinoma of the ovary (**Figure 1**). The detection of a *TP53* nonsense mutation supported the diagnosis of high-grade serous ovarian carcinoma (HGSOC) according to WHO classification (2). Integrating preoperative and postoperative pathology, immunohistochemistry, MRI, and FIGO staging (3), the final diagnosis was advanced ovarian cancer with implantation metastasis (stage IVA).

Postoperatively, the patient received standard first-line chemotherapy with intravenous paclitaxel (254 mg) and carboplatin (600 mg). A follow-up serum amylase test four weeks later showed normalization of amylase levels. The patient has adhered well to the treatment plan and remains under regular chemotherapy and monitoring.

## 3 Discussion

Ovarian cancer, one of the most common gynecological tumors of the female reproductive system, can occur at any age (4, 5). Due to its insidious onset, the disease is often associated with a persistently high mortality rate (6, 7). While some patients are diagnosed due to lower abdominal discomfort, most exhibit no apparent symptoms in the early stages or present with nonspecific manifestations that mask the underlying cause. Consequently, many are already at an advanced stage at the time of initial diagnosis.

### 3.1 Symptoms

The symptomatic presentation in this case was atypical and misleading. The patient had a relatively healthy medical history and no family history of tumors. Her initial symptoms consisted of chest tightness and exertional dyspnea—both nonspecific and attributable to a wide range of etiologies. Potential causes include acute or chronic cardiopulmonary diseases, pulmonary tuberculosis, pleural effusion secondary to lung tumors, or other pleural pathologies. When assessing pulmonary or neoplastic conditions, possible extrathoracic malignancies must also be carefully considered. A comprehensive diagnostic evaluation should incorporate demographic factors such as gender and age, along with a detailed medical history.

This case illustrates considerable diagnostic difficulty. Ovarian neoplasms are especially prone to being overlooked due to overlapping and nonspecific symptoms. The atypical presentation seen here can readily lead to a missed diagnosis of ovarian cancer, misdirect clinical judgment, and ultimately delay appropriate treatment.

### 3.2 Examination indicators

In this case, the patient showed a more pronounced elevation of amylase in the pleural fluid than in the serum or urine. Although elevated pleural fluid amylase is typically considered of pancreatic origin—since inflammation, tumors, or trauma can damage the pancreas or its ducts, leading to leakage of pancreatic fluid into the thoracic and abdominal cavities, which irritates the pleura and peritoneum and results in effusions—several findings argued against pancreatitis here. Notably, despite elevated pleural, serum, and urinary amylase, the more specific serum lipase was not increased, and the patient displayed no signs of an acute abdomen.

According to the Chinese Guidelines for the Diagnosis and Treatment of Acute Pancreatitis (2021) (8), acute pancreatitis requires at least two of the following:1) Persistent upper abdominal pain; 2) Serum lipase or amylase >3 times the upper limit of normal; 3) Imaging findings consistent with acute pancreatitis. The characteristic symptom is usually severe mid−epigastric pain radiating to the back (9). Based on the patient’s clinical presentation and imaging, acute or concurrent pancreatitis was considered unlikely. Therefore, the source of amylase elevation remained unclear. Subsequent staining of pleural effusion cells and ovarian tissue confirmed the diagnosis of high-grade serous adenocarcinoma of the ovary with thoracic metastasis. We thus speculate that the elevated amylase may originate from the ovarian tumor cells themselves.

### 3.3 Potential mechanisms linking elevated amylase levels to ovarian cancer

According to relevant literature, amylase genes *AMY1* (salivary-type) and *AMY2* (pancreatic-type) are also expressed in the lungs, trachea, ovaries, fallopian tubes, and cervix. Damage, inflammation, or tumors in these organs may activate these genes, leading to amylase synthesis and its secretion into the blood or body fluids, thereby elevating amylase levels (10). Alternatively, elevated amylase could be related to a paraneoplastic mechanism. Certain tumors, including small cell lung cancer and ovarian cancer, are known to secrete atypical bioactive substances as part of a paraneoplastic syndrome (11, 12). The increase in amylase may thus result from abnormal tumor cell metabolism or activation of relevant signaling pathways. In addition, inflammatory factors within the tumor microenvironment might stimulate adjacent tissues—such as mesothelial cells—to release amylase, although this mechanism requires further validation. Other possible explanations include metastatic invasion of the pancreas or pancreatic duct by ovarian cancer, leading to pancreatic-type amylase release, or tumor compression of lymphatic or vascular structures causing local ischemia or inflammation that indirectly promotes amylase release. Currently, however, evidence from this case is insufficient to support these latter hypotheses.

This case differs from previously reported ovarian cancers associated with hyperamylasemia. Among the approximately eight publications retrieved to date, most cases presented with abdominal symptoms, and hyperamylasemia was identified during the differential diagnosis (Ref. 11–17). In contrast, the initial symptom in this case was chest tightness—an atypical manifestation of ovarian cancer—rather than common signs such as lower abdominal pain. Notably, the amylase level in pleural fluid was markedly elevated at 4,995 U/L, significantly higher than that in serum (343 U/L; reference range: 35–135 U/L) and urine (1,513 U/L; reference range: 0–1,200 U/L). Such an insidious presentation accompanied by non-specific laboratory abnormalities substantially increases the risk of diagnostic delay.

Moreover, the patient presented with a massive left-sided pleural effusion in which the amylase level was markedly higher than in the serum. Immunohistochemical analysis of pleural effusion cells indicated a high-grade serous adenocarcinoma of ovarian origin, supporting the hypothesis that amylase may be produced by the ovarian cancer cells themselves. Notably, the patient’s preoperative amylase elevation returned to the normal range four weeks after surgery, consistent with findings reported by Yang et al. (13). The underlying mechanisms, however, require further investigation.

Elevated amylase is not specific to pancreatic disease. Besides acute or chronic pancreatitis, gallstones, cholangitis, intestinal perforation, or obstruction, hyperamylasemia has also been documented in multiple organ failure and diabetic ketoacidosis (14). Therefore, during differential diagnosis, the possibility of concurrent conditions contributing to amylase elevation should be carefully considered.

Currently, elevated amylase is not a routine diagnostic marker for ovarian cancer. However, an abnormal elevation may suggest an occult tumor, especially when accompanied by pleural or abdominal effusions. Although isolated cases have reported a link between ovarian cancer and increased serum amylase, large-scale clinical studies supporting this association are lacking (15).The mechanism behind amylase elevation in ovarian cancer may involve direct tumor secretion, paraneoplastic syndrome, or local microenvironmental changes. Nevertheless, the specific molecular pathways remain unclear and warrant further investigation.

The role of amylase as a potential tumor marker remains controversial. Elevated blood amylase has also been documented in myeloma, gastric cancer, lung cancer, and pheochromocytoma (16, 17), but the evidence mainly relies on case reports rather than large-scale studies. More extensive data are needed to clarify the specificity of amylase elevation and its relationship with tumor stage and pathological subtype. Future research could apply single-cell sequencing or proteomic technologies to elucidate the regulatory mechanisms of amylase gene expression in ovarian cancer cells. Meanwhile, multicenter studies should analyze both the incidence and prognostic significance of hyperamylasemia in ovarian cancer patients. Combining amylase with other established tumor markers (e.g., CA125 and HE4) may enable a more comprehensive clinical evaluation, particularly for early diagnosis and treatment monitoring.

This study has several limitations due to its retrospective design. Most notably, we lacked multi-site tissue sampling (e.g., from pancreas or parotid gland) and did not perform amylase isoenzyme typing. Pancreatic amylase is chiefly produced by pancreatic acinar cells, whereas salivary amylase has broader sources, including salivary glands, lungs, fallopian tubes, and certain tumors. Isoenzyme typing helps distinguish between pancreatic and salivary origins, thereby narrowing the diagnostic possibilities and improving specificity. Future studies should consider multi-site sampling and isoenzyme analysis to facilitate earlier differential diagnosis.

## 4 Conclusion

Due to the ovaries’ deep location within the pelvic cavity, early-stage ovarian cancer often presents with subtle and nonspecific symptoms, making it prone to being overlooked by clinicians. While chest tightness and shortness of breath are common clinical manifestations, their low specificity, resulting from multiple potential etiologies, requires physicians to differentiate and diagnose them thoroughly. For female patients with unusually elevated amylase levels, ovarian cancer should be considered as a possible diagnosis after excluding pancreatic-related diseases and comorbidities. When necessary, multi-site sampling should be conducted to identify the primary lesion.

## Data Availability

The original contributions presented in the study are included in the article/supplementary material. Further inquiries can be directed to the corresponding authors.

## References

[1] Vukovic A Kuna K Loncar Brzak B Vucicevic Boras V Separovic R Sekerija M . The role of salivary and serum CA125 and routine blood tests in patients with ovarian Malignancies. Acta Clin Croat. (2021) 60:55–62. doi: 10.20471/acc.2021.60.01.08, PMID: 34588722 PMC8305365

[2] WHO . Classification of tumours editorial board. WHO classification of tumors: female genital tumors. 5th edition. Lyon: International Agency for Research on Cancer (2020).

[3] Prat J D’Angelo E Espinosa I . Ovarian carcinomas: clinicopathologic and molecular features with comments on 2014 FIGO staging. Am J Surg Pathol. (2025) 49:e1e14. doi: 10.1097/PAS.0000000000002352, PMID: 39807827

[4] Cao W Chen HD Yu YW Li N Chen WQ . Changing profiles of cancer burden worldwide and in China: a secondary analysis of the global cancer statistics 2020. Chin Med J (Engl). (2021) 134:783–91. doi: 10.1097/CM9.0000000000001474, PMID: 33734139 PMC8104205

[5] Ledermann JA Matias-Guiu X Amant F Concin N Davidson B Fotopoulou C . ESGO-ESMO-ESP consensus conference recommendations on ovarian cancer: pathology and molecular biology and early, advanced and recurrent disease. Ann Oncol. (2024) 35:248–66. doi: 10.1016/j.annonc.2023.11.015, PMID: 38307807

[6] Yuan MW Feng YS Zhao XL Hu SY Zhao FH . Incidence trends of breast cancer and reproductive system cancers among Chinese women from 2006 to 2017. Chin J Epidemiol. (2024) 45:647–55. doi: 10.3760/cma.j.cn112338-20231103-00271

[7] Webb PM Jordan SJ . Global epidemiology of epithelial ovarian cancer. Nat Rev Clin Oncol. (2024) 21:389–400. doi: 10.1038/s41571-024-00881-3, PMID: 38548868

[8] Pancreatic Surgery Group Branch of Surgery Chinese Medical Association . Chinese guidelines for the diagnosis and treatment of acute pancreatitis (2021). Zhejiang Pract Med. (2021) 26:511–9. doi: 10.16794/j.cnki.cn33-1207/r.2021.06.003

[9] Mao EQ Che ZQ . Interpretation of the expert consensus on emergency diagnosis and treatment of acute pancreatitis. J Clin Emerg. (2024) 25:325–8. doi: 10.13201/j.issn.1009-5918.2024.07.001

[10] Tian Y Cao XS . Ovarian cancer with elevated amylase: A case report. Chin J Interv Imaging Ther. (2020) 17:83. doi: 10.13929/j.issn.1672-8475.2020.02.005

[11] Song J Fan L . Small cell lung cancer with dual paraneoplastic syndromes: a case report. Case Rep Oncol. (2025) 18:159–68. doi: 10.1159/000542763, PMID: 39980499 PMC11781813

[12] Guo S Lv H Yan L Rong F . Hyperamylasemia may indicate the presence of ovarian carcinoma: a case report. Med (Baltimore). (2018) 97:e13520. doi: 10.1097/MD.0000000000013520, PMID: 30544453 PMC6310503

[13] Yang B Yang YX . Malignant ovarian tumor with hyperamylasemia and hyperamylasuria: A case report. Int J Obstet Gynecol. (2019) 46:161–2.

[14] Burden S Poon AS Masood K Didi M . Hyperamylasaemia: pathognomonic to pancreatitis. BMJ Case Rep. (2013) 2013. doi: 10.1136/bcr-2013-009567, PMID: 24132440 PMC3822262

[15] Jie Y Li J Man CF Fan Y . Ovarian cancer with intestinal wall invasion and hyperamylasemia: a case report. Front Oncol. (2024) 14:1299226. doi: 10.3389/fonc.2024.1299226, PMID: 38406808 PMC10884171

[16] Casadei GA Mariotti M Lucchesi A Pini S Valgiusti M Bravaccini S . Paraneoplastic lipase and amylase production in a patient with small-cell lung cancer: case report. BMC Cancer. (2016) 16:118. doi: 10.1186/s12885-016-2167-7, PMID: 26887807 PMC4758001

[17] Benedetti G Rastelli F Damiani S Calandri C Crino L . Challenging problems in Malignancy: case 1. Presentation of small-cell lung cancer with marked hyperamylasemia. J Clin Oncol. (2004) 22:3826–8. doi: 10.1200/JCO.2004.11.099, PMID: 15365080

